# Supplementary material for: Association of Sonographic Sarcopenia and Falls in Older Adults Presenting to the Emergency Department
Source: J Clin Med. 2023 Feb 4;12(4):1251. doi: 10.3390/jcm12041251 (PMC9968231; doi:10.3390/jcm12041251)
Supplement: Supplementary file 1 [file jcm-12-01251-s001.zip › jcm-2138498-supplementary.pdf]

Supplementary Table S1

Correlation matrix

Estimated Correlation Matrix								
**Parameter**	**Intercept**	**age**	**sex**	**Biceps**	**Thight**	**Grip Max**	**CCI > 6**	**Polypharmacy**
**Intercept**	1.0000	-0.8052	-0.3846	-0.3528	-0.1932	-0.3134	-0.1108	-0.2962
**Age**	-0.8052	1.0000	0.0717	0.1612	0.0446	0.3102	0.0021	-0.0883
**Sex**	-0.3846	0.0717	1.0000	0.3003	0.0823	-0.0522	0.0793	-0.0702
**Biceps**	-0.3528	0.1612	0.3003	1.0000	-0.4784	-0.0443	0.1471	0.0197
**Thigh**	-0.1932	0.0446	0.0823	-0.4784	1.0000	-0.0500	-0.0765	-0.0549
**Grip Max**	-0.3134	0.3102	-0.0522	-0.0443	-0.0500	1.0000	0.1797	0.0781
**CCI > 6**	-0.1108	0.0021	0.0793	0.1471	-0.0765	0.1797	1.0000	0.0364
**Polypharmacy**	-0.2962	-0.0883	-0.0702	0.0197	-0.0549	0.0781	0.0364	1.0000

Supplementary Table S2

Maximum Likelihood Estimates

Analysis of Maximum Likelihood Estimates					
**Parameter**	**DF**	**Estimate**	**Standard Error**	**Wald Chi-Square**	**Pr > ChiSq**
**Intercept**	1	0.5063	2.2132	0.0523	0.8190
**Age**	1	0.000497	0.0199	0.0006	0.9801
**Sex**	1	0.0600	0.3346	0.0321	0.8578
**Biceps**	1	0.4444	0.2838	2.4523	0.1174

Analysis of Maximum Likelihood Estimates					
**Parameter**	**DF**	**Estimate**	**Standard Error**	**Wald Chi-Square**	**Pr > ChiSq**
**Thigh**	1	-0.9361	0.4047	5.3511	0.0207
**Grip Max**	1	-2.0466	1.2945	2.4997	0.1139
**Thigh & Grip Max**	1	0.5300	0.4344	1.4886	0.2224
**CCi > 6**	1	-0.1130	0.3831	0.0870	0.7681
**Polypharmacy**	1	1.2333	0.8477	2.1169	0.1457
